# Supplementary material for: Cell-free assays reveal the HIV-1 capsid protects reverse transcripts from cGAS
Source: bioRxiv. 2024 Apr 23:2024.04.22.590513. Preprint. [Version 2] doi: 10.1101/2024.04.22.590513 (PMC11071359; doi:10.1101/2024.04.22.590513)
Supplement: Supplementary file 1 [file media-1.pdf]

Supplementary Information for:

Cell-free assays reveal the HIV-1 capsid protects reverse transcripts from cGAS

Tiana M. Scott[1], Lydia M. Arnold[1], Jordan A. Powers[1], Delaney A. McCann[1], Devin E. Christensen[2], Wen Zhou[3], Rachel M. Torrez[2], Janet H. Iwasa[2], Philip J. Kranzusch[4,5], Wesley I. Sundquist[2], Jarrod S. Johnson[1,6]*

[1]Division of Microbiology and Immunology, Department of Pathology, University of Utah School of Medicine; Salt Lake City, UT 84112, USA

[2]Department of Biochemistry, University of Utah School of Medicine; Salt Lake City, UT 84112, USA

[3]Department of Immunology and Microbiology, School of Life Sciences, Southern University of Science and Technology; Shenzhen, Guangdong 518055, China

[4]Department of Microbiology, Harvard Medical School; Boston, MA 02115, USA

[5]Department of Cancer Immunology and Virology, Dana-Farber Cancer Institute; Boston, MA 02115, USA

*Corresponding author
Email: jarrod.johnson@path.utah.edu

Supplementary Information includes:

S1 Table. Reagents used in this study.

S1 Fig. Capsid stability can be affected by heat or CA mutations.

S2 Fig. Cell-free sensing of HIV-1 requires virion permeabilization, magnesium, NTPs, and cGAS.

S3 Fig. Specific CA mutations confer resistance to LEN.

S4 Fig. PQBP1 does not affect cGAS activity *in vitro* and is not essential in cells.

S1 Movie. Model for LEN-mediated disruption of HIV-1 cores and cGAS-STING activation. (separate file)

A molecular animation depicting an HIV-1 viral core in the cytoplasm of myeloid cells, with reverse transcription proceeding inside an intact or largely intact capsid. In the presence of lenacapavir, accumulated reverse transcripts can become exposed by drug-induced capsid rupturing, enabling viral DNA detection and downstream activation of interferon production via the cGAS-STING-IRF3 pathway.

S1 Table. Reagents used in this study.

REAGENT	Source	Catalogue #
Antibodies		
cGAS	Cell Signaling	Cat# 79978<br>RRID:AB_2905508
STING	Cell Signaling	Cat# 13647<br>RRID:AB_2732796
IRF3	Cell Signaling	Cat# 11904<br>RRID:AB_2722521
PQBP1	Proteintech	Cat# 16264-1-AP<br>RRID:AB_10792928
ISG15-PE	R&D Systems	Cat# IC8044P
SIGLEC1-647	BD Biosciences	Cat# 565295<br>RRID:AB_2739163
Actin	BD Biosciences	Cat# 612656<br>RRID:AB_2289199
IRDye 680RD Donkey anti-Mouse fluorescent antibody	LI-COR	Cat# 926-68072<br>RRID:AB_10953628
IRDye 800CW Goat anti-Rabbit fluorescent antibody	LI-COR	Cat# 926-32211<br>RRID:AB_621843
Bacterial and virus strains		
Stbl3 competent *E. coli*	Thermo Fisher	Cat# C737303
BL21-CodonPlus (DE3)-RIPL *E. coli*	Agilent	Cat# 230280
BL21-CodonPlus (DE3)-RIL *E. coli*	Agilent	Cat# 230245
HIV-1 SG3ΔENV	NIH AIDS Reagent Program	Addgene plasmid #11051
HIV-1 SG3ΔENV RT D185A	Christensen et al., 2020	Addgene plasmid #145782
HIV-1 SG3ΔENV CA E45A	Christensen et al., 2020	Addgene plasmid #145783
HIV-1 SG3ΔENV CA Q63/67A	Christensen et al., 2020	Addgene plasmid #145784
HIV-1 SG3ΔENV CA M66I	Christensen et al., 2020	Addgene plasmid #149687
HIV-1 SG3ΔENV CA Q67H/N74D	This study	Addgene plasmid #217442
HIV-1-GFPΔENV	This study	Addgene plasmid #217437
HIV-1-GFP CA E45A	This study	Addgene plasmid #217438
HIV-1-GFP CA Q63/67A	This study	Addgene plasmid #217439
HIV-1-GFP CA M66I	This study	Addgene plasmid #217440
HIV-1-GFP CA Q67H/N74D	This study	Addgene plasmid #217441
pSIV3+	Mangeot et al., 2000	N/A
Chemicals, peptides, and recombinant proteins		
Melittin	Sigma Aldrich	Cat# M2272-5MG
IP6	Sigma Aldrich	Cat# I5125-50G
rATP	Promega	Cat# E6011
rGTP	Promega	Cat# E6031
rCTP	Promega	Cat# E6041
rUTP	Promega	Cat# E6021
dATP	Promega	Cat# U120D
dGTP	Promega	Cat# U121D
dCTP	Promega	Cat# U122D
dTTP	Promega	Cat# U123D

HEPES	Gibco	Cat# 15630080
Phosphate Buffer Saline (PBS)	Gibco	Cat# 14190250
EDTA 0.5M	Invitrogen	Cat# AM9260G
Penicillin-Streptomycin	Thermo Fisher	Cat# 15140-122
Chloramphenicol	Thermo Fisher	Cat# B20841.22
2-Mercaptoethanol	Thermo Fisher	Cat# 21985023
MEM non-essential amino acids	Thermo Fisher	Cat# 11140050
RPMI 1640 medium	Thermo Fisher	Cat# 11875-119
DMEM	Thermo Fisher	Cat# 11995073
Ficoll-Paque Plus	GE Healthcare	Cat# 17-1440-02
Fetal Bovine Serum	Gibco	(Lot #1982147)
Fetal Bovine Serum	Gibco	(Lot #1982147)
Recombinant Human IL-4	Miltenyi Biotech	Cat# 130-093-922
Recombinant Human GM-CSF	Miltenyi Biotech	Cat# 130-093-867
poly-L-lysine hydrobromide	MP Biomedicals	Cat# 0219454405
Polyethylenimine “Max”, (Mw 40,000) - High Potency Linear PEI	Polysciences, Inc.	Cat# 24765-1
Paraformaldehyde	Electron Microscopy Sciences	Cat# 15713-S
Polybrene	Sigma	Cat# TR-1003-G
Puromycin	Invivogen	Cat# ant-pr-1
Halt protease and phosphatase inhibitor	Thermo Fisher	Cat# 78441
Efavirenz	Selleck Chemicals	S4685
Elvitegravir	Selleck Chemicals	S2001
Lenacapavir	MCE	HY-111964
GS-CA1	Gilead	N/A
PF-74 (PF-3450074)	MCE	HY-120072
BOLT transfer buffer (20X)	Invitrogen	BT00061
Novex NuPAGE MOPS SDS Running Buffer (20X)	Invitrogen	NP0001
NuPAGE LDS Sample Buffer (4X)	Invitrogen	NP0007
Benzonase	Sigma	Cat# E1014
Subtilisin Protease from Bacillus	Sigma	Cat# P5380
PMSF	Roche	Cat# 10837091001
NaCl	Thermo Fisher	Cat# BP358-10
Tris-HCl	Thermo Fisher	Cat# 228030051
Tris-base	Thermo Fisher	Cat# BP152-500
$MgCl_2$	Thermo Fisher	Cat# 41341-5000
Isopropyl ß-D-1-thiogalactopyranoside	GoldBio	Cat# I2481C50
2xYT media	Sigma	Cat# Y1003
Imidazole	Thermo Fisher	Cat# AC122020020
Lysozyme	GoldBio	Cat# L-040-100
Pepstatin	Sigma	Cat #11524488001
Leupeptin	Sigma	Cat #L2884
Aprotinin	Sigma	Cat# 10981532001
DNase I	GoldBio	Cat# D-300-500
His_6-ULP1 protease	Produced in-house	Lao et al. 2018, J. Biol. Chem.
TCEP	Sigma	Cat# C4706
Dithiothreitol (DTT)	Roche	Cat# 10197777001
Ni-NTA resin	Qiagen	Cat# 30210
Casamino acids	Gibco	Cat# 223050
cOmplete His-Tag purification beads	Roche	Cat# 5893682001

**Oligonucleotides**		
MSSS-FWD (early): AACCCACTGCTTAAGCCTCA	Christensen et al.	N/A
MSSS-REV (early): ACCAGAGTCACACAACAGACG	Christensen et al.	N/A
FST-FWD (intermediate): AGCCGCCTAGCATTTCATCA	Christensen et al.	N/A
FST-REV (intermediate): CCAGCGGAAAGTCCCTTGTA	Christensen et al.	N/A
Late RT-FWD: TGTGTGCCCGTCTGTTGTGT	Christensen et al.	N/A
Late RT-REV: CTTCAGCAAGCCGAGTCCTG	Christensen et al.	N/A
cGAS gRNA target: GAGGCCGCCCTGCCTAAGGC	Johnson et al.	N/A
STING gRNA target: CCCCGTGACCCCTGGGACAC	Johnson et al.	N/A
IRF3 gRNA target: CGTGCGGCTCTTGTTCACCC	Johnson et al.	N/A
PQBP1 KO1 gRNA target: GTCTGCAGCGCAACGGGCAG	This study	N/A
PQBP1 KO2 gRNA target: GGATGCCTCTCTTGGCCAAG	This study	N/A
**Recombinant DNA**		
psPAX2	Gift from Didier Trono	Addgene plasmid #12260
pCMV-VSV-G	Gift from Bob Weinberg	Addgene plasmid #8454
pCA528	Gift from Wes Sundquist	DNASU plasmid pCA528
pCA528-huPQBP1	This study	Addgene plasmid #217449
lentiCRISPR v2	Gift from Feng Zhang	Addgene plasmid #52961
lentiCRISPR control (LCV2) empty vector minus stuffer	Johnson et al., 2018	Addgene plasmid #217443
lentiCRISPR cGAS	Johnson et al., 2018	Addgene plasmid #217444
lentiCRISPR STING	Johnson et al., 2018	Addgene plasmid #217445
lentiCRISPR IRF3	Johnson et al., 2018	Addgene plasmid #217446
lentiCRISPR PQBP1 KO1	This study	Addgene plasmid #217447
lentiCRISPR PQBP1 KO2	This study	Addgene plasmid #217448
**Software**		
GraphPad Prism 9.0	GraphPad	https://www.graphpad.com/scientific-software/prism/
FlowJo 10.0	FlowJo LLC	https://www.flowjo.com/
Image Studio Lite 5.5	LI-COR	https://www.licor.com/bio/image-studio-lite/
Morpheus	Broad Institute	https://software.broadinstitute.org/morpheus/
Illustrator	Adobe	https://www.adobe.com/products/illustrator
BioRender	BioRender	https://www.biorender.com/
**Other Reagents**		
LS Columns	Miltenyi Biotec, Inc	130-042-401
CD14 MicroBeads, human	Miltenyi Biotec, Inc	130-050-201
QuadroMACS Separator	Miltenyi Biotec, Inc	130-090-976
ProFlex PCR System	Thermo Fisher	A41182

QuantStudio 3 Real-Time PCR System	Applied Biosystems	A28137
Attune NxT Flow Cytometer	Thermo Fisher	A24858
Attune NxT Autosampler	Thermo Fisher	4473928
Odyssey CLx Imager	LI-COR	CLX-2063
SpectraMax ID.5 Plate Reader	Molecular Devices	Cat #76175-288
BioTek Synergy Neo2 Plate Reader	Agilent	BTNEO2
SW 32 Ti Rotor, Swinging Bucket	Beckman	Cat# 369694
0.45µm syringe filters	Corning	Cat# 28200-026
Optima Ultracentrifuge	Beckman	Model: LE-80k
Bolt 4-12% Bis-Tris Plus Gels	Invitrogen	NW04125BOX
HiTrap Heparin HP 5 mL	Cytiva	Cat #17040701
HiTrap Q HP 5 mL	Cytiva	Cat #17115401
Superdex 75, 120 mL;16/600	Cytiva	Cat# 28989333
Thinwall Polyallomer, Konical Tubes; 25 x 89 mm	Beckman	Cat# 358126
Open-Top Thinwall Ultra-Clear Tube, 25 x 89 mm	Beckman	Cat# 344058
2',3'-cGAMP ELISA Kit	Arbor Assays	Cat# K067-H5
LIVE/DEAD® Fixable Violet Dead Cell Stain Kit	Molecular Probes	L34955
BCA protein assay, reducing agent compatible	Thermo Fisher	Cat# PI23252
p24 ELISA	Xpress Bio	XB-1000
PureLink HiPure Plasmid Maxiprep Kit	Invitrogen	K210007
QIAquick PCR Purification Kit	Qiagen	Cat# 28106
Cytofix/Cytoperm Fixation/Permeabilization Kit	BD Biosciences	554714
Human leukocytes from normal donors (deidentified)	ARUP Components	N/A

S1 Fig.

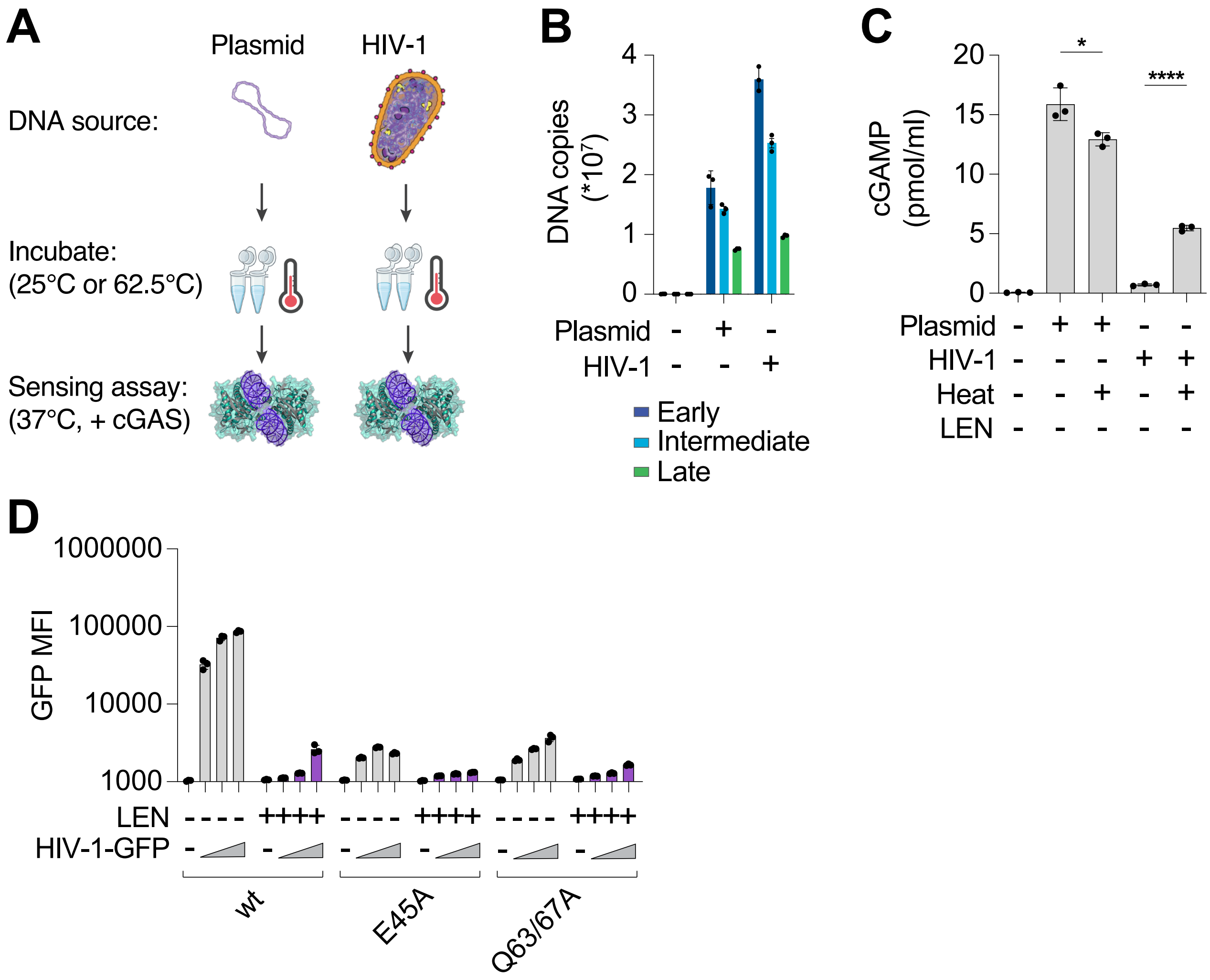

A
Plasmid
HIV-1
DNA source:
Incubate:
(25°C or 62.5°C)
Sensing assay:
(37°C, + cGAS)
B
DNA copies
(*10^7)
Plasmid - + -
HIV-1 - - +
Early
Intermediate
Late
C
cGAMP
(pmol/ml)
*
****
Plasmid - + + - -
HIV-1 - - - + +
Heat - - + - +
LEN - - - - -
D
GFP MFI
1000000
100000
10000
1000
LEN - - - - + + + + - - - - + + + + - - - - + + + +
HIV-1-GFP
wt
E45A
Q63/67A

S1 Fig. Capsid stability can be affected by heat or CA mutations.

(A) Schematic depicting heat treatment of plasmid or ERT samples prior to cell-free cGAS sensing assays.

(B) Copy number of plasmid DNA or viral DNA from ERT reactions incubated under identical conditions to be taken through limited heat treatment.

(C) Cell-free sensing assay (related to panels A&B) comparing the ability of plasmid DNA and HIV-1 ERT samples to activate recombinant cGAS. Heat treated samples were incubated briefly (20 s) at 62.5ºC before assaying with cGAS under standard conditions. Heat treatment slightly decreased the ability of plasmid DNA to activate cGAS (possibly through partial denaturation and/or aggregation of the DNA into a less accessible structure). In contrast, heat treatment of ERT products robustly increased cGAS activity.

(D) GFP mean fluorescence intensity measured by flow cytometry in THP-1 monocytic cells that were infected for 48 h with HIV-1-GFP, the capsid stabilized CA mutant E45A, or the destabilized mutant Q63/67A at matched virus doses (12.5, 25, and 50 nM p24). Where indicated, LEN (10 nM) was added at the time of infection.

For (C), statistics were calculated using an unpaired t test to compare plasmid or HIV-1 samples: $p<0.05$: *, $p<0.0001$: ****. Graphs show mean ± SD from three samples from a representative experiment, selected from two independent experiments.

S2 Fig.

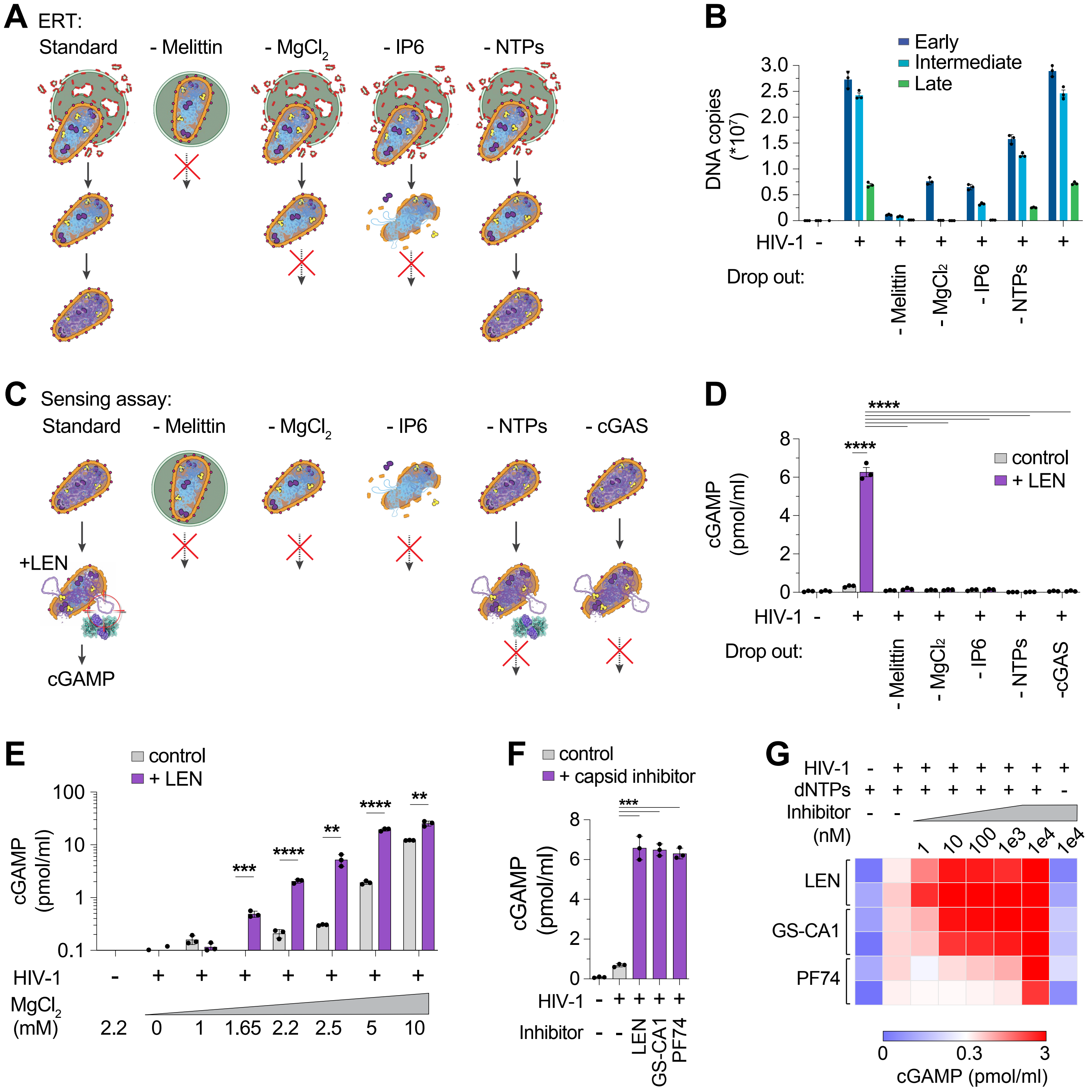
A
ERT:
Standard
- Melittin
- MgCl2
- IP6
- NTPs
B
Early
Intermediate
Late
DNA copies (*10^7)
3.0
2.5
2.0
1.5
1.0
0.5
0
HIV-1
Drop out:
- Melittin
- MgCl2
- IP6
- NTPs
C
Sensing assay:
Standard
- Melittin
- MgCl2
- IP6
- NTPs
- cGAS
+LEN
cGAMP
D
****
cGAMP (pmol/ml)
control
+ LEN
HIV-1
Drop out:
- Melittin
- MgCl2
- IP6
- NTPs
-cGAS
E
control
+ LEN
cGAMP (pmol/ml)
100
10
1
0.1
***
****
**
****
**
HIV-1
MgCl2 (mM)
2.2
0
1
1.65
2.2
2.5
5
10
F
control
+ capsid inhibitor
***
cGAMP (pmol/ml)
HIV-1
Inhibitor
LEN
GS-CA1
PF74
G
HIV-1
dNTPs
Inhibitor (nM)
1
10
100
1e3
1e4
1e4
LEN
GS-CA1
PF74
0
0.3
3
cGAMP (pmol/ml)

S2 Fig. Cell-free sensing of HIV-1 requires virion permeabilization, magnesium, NTPs, and cGAS.

(A) Schematic illustrating that under standard ERT reaction conditions, viral RNA is converted into DNA inside an intact or largely intact capsid. When key components are omitted from the reaction, the ERT reaction is inhibited at the indicated steps.

(B) qPCR measurements of DNA copy numbers from ERT reactions under standard conditions compared to reactions performed in the absence of melittin, $MgCl_2$, IP6, or NTPs.

(C) Models for cell-free sensing reactions corresponding to ERT conditions depicted in (A). Core disruption with LEN permits cGAS sensing of RT products under standard reactions conditions. Sensing is blocked at the indicated steps when specific reaction components are removed.

(D) Cell-free cGAS sensing assay performed with ERT samples shown in (B) under standard conditions compared to drop out conditions with or without LEN (100 nM).

(E) Cell-free sensing assay showing sensitivity to $MgCl_2$ concentration. Standard HIV-1 ERT samples were aliquoted into cGAS reactions with the indicated amounts of $MgCl_2$, and with or without LEN (100 nM).

(F) Cell-free sensing assay of samples incubated after ERT in the presence or absence of LEN (100 nM), GS-CA1 (100 nM), or PF74 (10 µM).

(G) Heat map showing dose-dependent increase in cGAS activity for the indicated capsid inhibitors. Two replicates shown from one of three independent experiments. Statistics were calculated using a one-way ANOVA with Tukey's multiple comparisons test in (D,F) or an unpaired t test for each concentration of $MgCl_2$ in (E): $p<0.01$: **, $p<0.001$: ***, $p<0.0001$: ****. Graphs depict mean ± SD from three samples from a representative experiment, selected from three independent experiments.

S3 Fig.

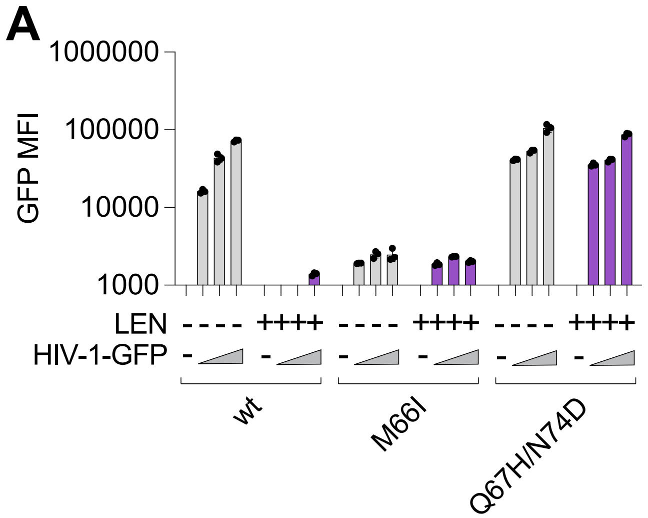
A
1000000
100000
10000
1000
GFP MFI
LEN
- - - -
++++
- - - -
++++
- - - -
++++
HIV-1-GFP
-
-
-
-
-
-
wt
M66I
Q67H/N74D

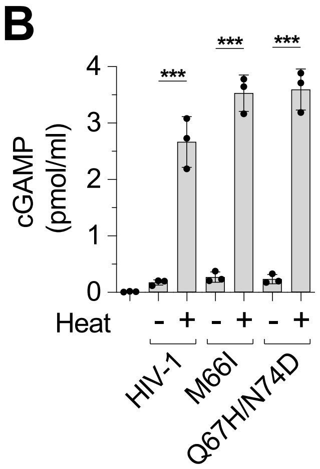
B
***
***
***
4
3
2
1
0
cGAMP (pmol/ml)
Heat
-
+
-
+
-
+
HIV-1
M66I
Q67H/N74D

S3 Fig. Specific CA mutations confer resistance to LEN.

(A) Mean fluorescence intensity of GFP measured by flow cytometry in THP-1 monocytic cells infected for 48 h with HIV-1-GFP compared to LEN-resistant CA mutants M66I and Q67H/N74D at matched virus doses (12.5, 25, and 50 nM p24). Where indicated, LEN (10 nM) was added at the time of infection.

(B) Cell-free sensing assay of the indicated ERT samples under standard conditions or after limited heat treatment at 62.5°C.

Statistics were calculated using a one-way ANOVA with Tukey's multiple comparisons test: $p<0.001$: ***.

Graphs depict mean ± SD from three samples from a representative experiment, selected from 2 (A) or 3 (B) independent experiments.

S4 Fig.

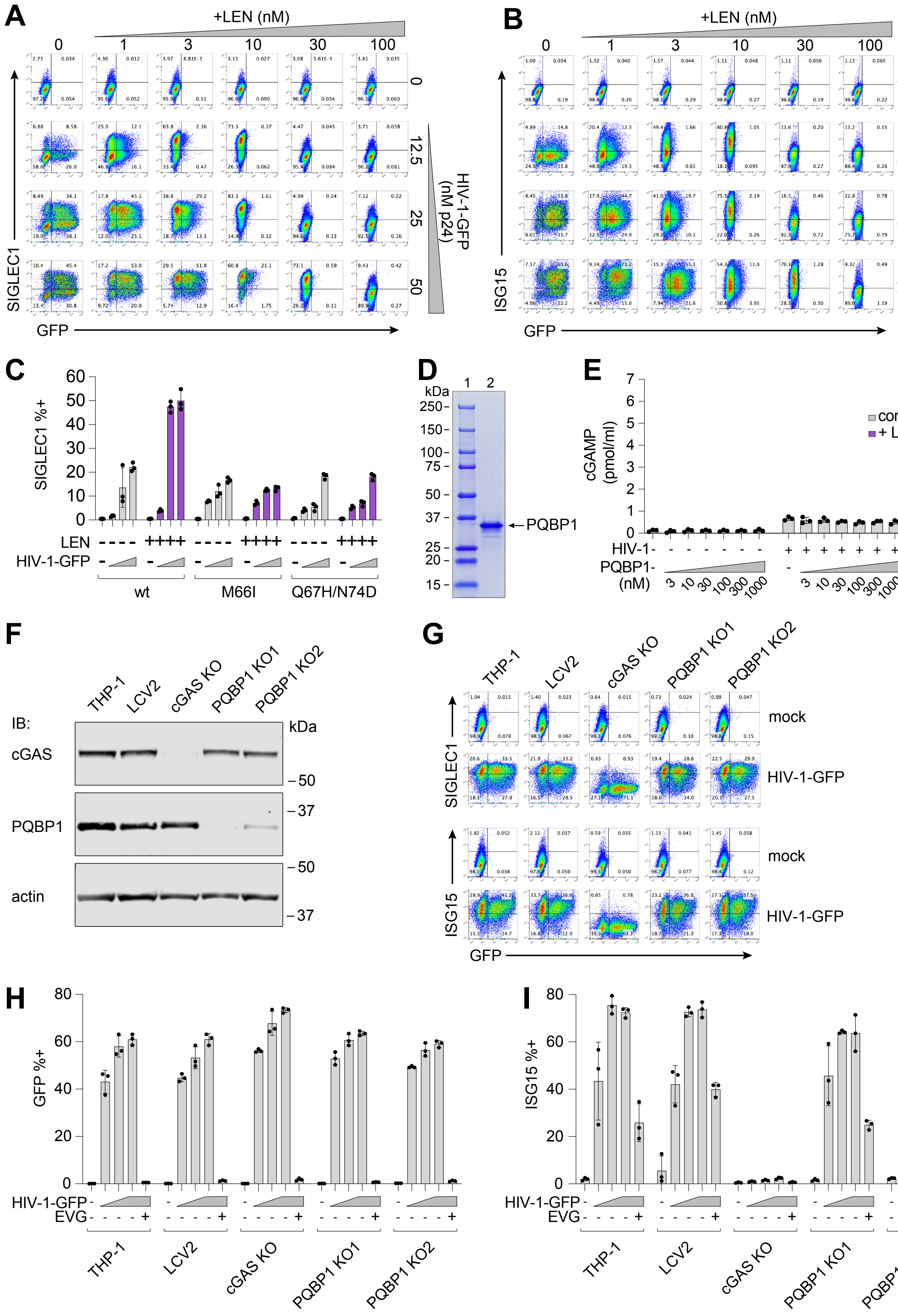
A
+LEN (nM)
0
1
3
10
30
100
HIV-1-GFP (nM p24)
12.5
25
50
SIGLEC1
GFP
B
ISG15
C
SIGLEC1 %+
LEN
HIV-1-GFP
wt
M66I
Q67H/N74D
D
kDa
PQBP1
E
cGAMP (pmol/ml)
control
+ LEN
HIV-1
PQBP1 (nM)
F
THP-1
LCV2
cGAS KO
PQBP1 KO1
PQBP1 KO2
IB:
cGAS
PQBP1
actin
G
mock
HIV-1-GFP
H
GFP %+
EVG
I
ISG15 %+

S4 Fig. PQBP1 does not affect cGAS activity *in vitro* and is not essential in cells.

(A-B) Flow cytometry of THP-1 monocytic cells infected for 48 h with a range of HIV-1-GFP doses (12.5, 25, and 50 nM p24) and increasing amounts of LEN. GFP is displayed on the x-axis and either SIGLEC1 (A) or ISG15 (B) are shown on the y-axis. Plots represent one of three replicates, which are averaged in the heat maps shown in Fig. 4C.

(C) Percent SIGLEC1-positive cells measured by flow cytometry. THP-1 monocytic cells were infected for 48 h with HIV-1-GFP compared to LEN-resistant CA mutants M66I and Q67H/N74D at matched doses (12.5, 25, and 50 nM p24). LEN (10 nM) was added at the time of infection.

(D) SDS-PAGE with Coomassie staining showing a molecular weight ladder (lane 1) and purified recombinant human PQBP1 protein (lane 2).

(E) Cell-free sensing assay to determine cGAS activity in the presence or absence of PQBP1. Purified PQBP1 was incubated at increasing concentrations with recombinant cGAS alone, or together with HIV-1 samples aliquoted from standard ERT reactions. Cell-free sensing assay reactions were performed under standard conditions. LEN (100 nM) was included as a positive control for viral capsid disruption.

(F) Immunoblots of THP-1 lysates after lentiCRISPR editing, depicting KO of cGAS or PQBP1 (two independent gRNAs targeting PQBP1), relative to a non-targeting vector control (LCV2).

(G) Flow cytometry of lentiCRISPR-modified THP-1 cells infected with HIV-1-GFP for 48 h (50 nM p24), depicting GFP expression on the x-axis and either SIGLEC1 or ISG15 on the y-axis.

(H-I) Flow cytometry data from THP-1 cells infected with a range of HIV-1-GFP doses (12.5, 25, and 50 nM p24) for 48 h, showing %GFP+ (H) or %ISG15+ (I). To evaluate whether cGAS or PQBP1 have roles in sensing early steps in the virus life cycle, we also tested HIV-1-GFP (50 nM p24) in the presence of the integration inhibitor elvitegravir (EVG, 1 μM). All graphs show mean ± SD from three samples from a representative experiment, selected from two independent experiments.
